# Cut-off Scale and Complex Formation in Density Functional Theory Computations of Epoxy-Amine Reactivity

**DOI:** 10.1021/acsomega.1c03229

**Published:** 2021-10-25

**Authors:** Pekka V. Laurikainen, Essi L. Sarlin

**Affiliations:** Faculty of Engineering and Natural Sciences, Tampere University, P. O. Box 589, FI-33014 Tampere, Finland

## Abstract

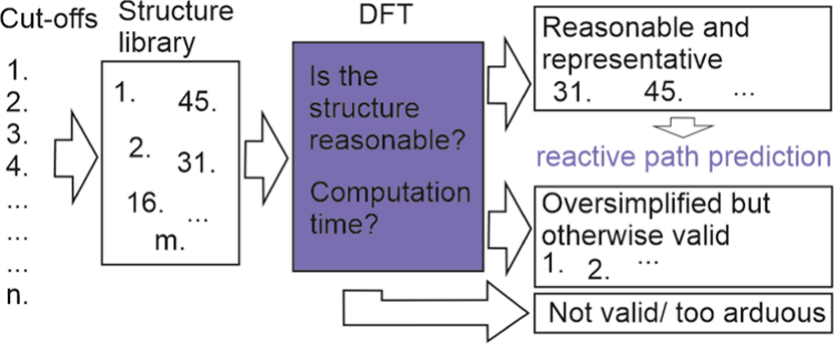

Most of the properties of epoxy resins are tied to their degree of cross-linking, making understanding the reactivity of different epoxy systems a crucial aspect of their utilization. Here, epoxy-amine reactivity is studied with density functional theory (DFT) at various cut-off levels to explore the suitability of the method for estimating the reactivity of specific epoxy systems. Although it is common to use minimal structures in DFT to reduce computational cost, the results of this study highlight the important role of hydrogen bonding and other noncovalent interactions in the reactivity. This is a promising result for differentiating the most probable reactive paths for different resin systems. The significance of amine groups as a potential source of catalyzing H-bonds was also explored and, while not quite as effective as a catalyst as a hydroxyl group, a clear catalyzing effect was observed in the transition state energies. Unfortunately, the added complexity of a more representative reactive system also results in increased computational cost, highlighting the need for proper selection of structural cutoffs.

## 1 Introduction

For many epoxy applications, in-depth knowledge of the resin system chemistry is essential. The basic reactions of epoxy resins can be considered well understood, but the kinetics of curing,^1−3^ ageing behavior,^4,5^ polymer network formation,^3,6,7^ and options for tailoring the structure and properties^8−10^ remain subjects of active study in the field. The studies of the reactivity at the scale of individual molecules, bonds, and small differences between spatial isomers are only possible through *ab initio* or the computationally less arduous density functional theory (DFT) computations.^11−13^ The performance and approximations offered by modern DFT implementations enable approaching the scale of full resin molecules, expanding the potential user base of the methods also toward materials science and chemical engineering.

Research on epoxy-amine reactivity commonly relates to different modifications of the base reaction, for example, the catalysis provided by hydrogen bonding.^14^ The possible effects of hydrogen bonds (H-bonds) are twofold: H-bonding can lower the reactive barrier (catalysis) or stabilize the intermediate structure, causing a retardation effect.^13^ Raman and Palmese^13^ studied the latter by adding tetrahydrofuran into an epoxy-amine mixture. A similar retardation could also result from H-bond acceptors in the structure of the reagents, such as oxygen bridges in the epoxy and/or amine backbone. The catalyzing H-bonds are mostly considered to form with the hydroxyl groups formed during the addition reaction between the primary amine and the epoxide ring.^11−15^ Most studies ignore the possible role of another H-bond donor—the amine groups themselves—likely due to the lower reactive barriers reported for hydroxyl group-catalyzed reactive paths.^11^ However, the abundance of available amine end-groups in epoxy-amine systems, especially in the early stages of the curing reactions, makes the amine groups a promising source of H-bonds.

In addition to H-bonding, epoxy-amine reactions are affected by inductive effects from the various chemical groups connected to the reactive center.^14^ One example is the α-effect.^16^ The α-effect is strong in methylated amines^17^ and can, therefore, contribute to the results of computations of simplified structures. At least, if correctly predicted by the computation. The inductive effects are much weaker for larger structures^18^ but, nevertheless, need to be considered in the total effect substituents have on the energy of the reaction and when estimating how changing the scale of the model system affects the computational results.

In this study, epoxy-amine reactions are studied computationally using modern DFT methods. The energy levels of the structures along the reactive path are computed at varying structural cutoffs. The aim is to explore the accuracy versus computational cost relation for a model epoxy-amine system and to see the effects various neighboring chemical groups can have on the reactive path. This should allow the qualitative prediction of the most probable reactive path for a given cutoff and offer insights into the complex role of noncovalent bonding in stabilizing the structures along the reactive path. The different cut-off structures are manually built to represent commonly encountered epoxy-amine reactive systems with increasing complexity. For example, all epoxy structures are simplified cutoffs of diglycidylether of Bisphenol-A (DGEBA).

## 2 Computational Methods

The numerical computation was performed using the Schrödinger Materials Science Suite (Schrödinger Inc., New York, USA) software package (version 2020-4)—most prominently the Jaguar (version 11.0) DFT program.^19^ The long-range corrected hybrid nonlocal B3LYP-D3 theory level was used for all DFT computations. The functional has been shown to give a good combination of accuracy and computational efficiency with many types of chemical structures and noncovalent bonding cases.^20,21^ The M06-2X and ωB97X-D functionals along with B3LYP-D3 with Becke–Johnson damping^22^ were tested in single point energy (SPE) computations for comparison. This part of the discussion is presented in the Supporting Information. The basis set was varied depending on the computational task. The 6-31G** basis set was used for the transition state searches and other initial structural optimizations.

**Figure 1:**
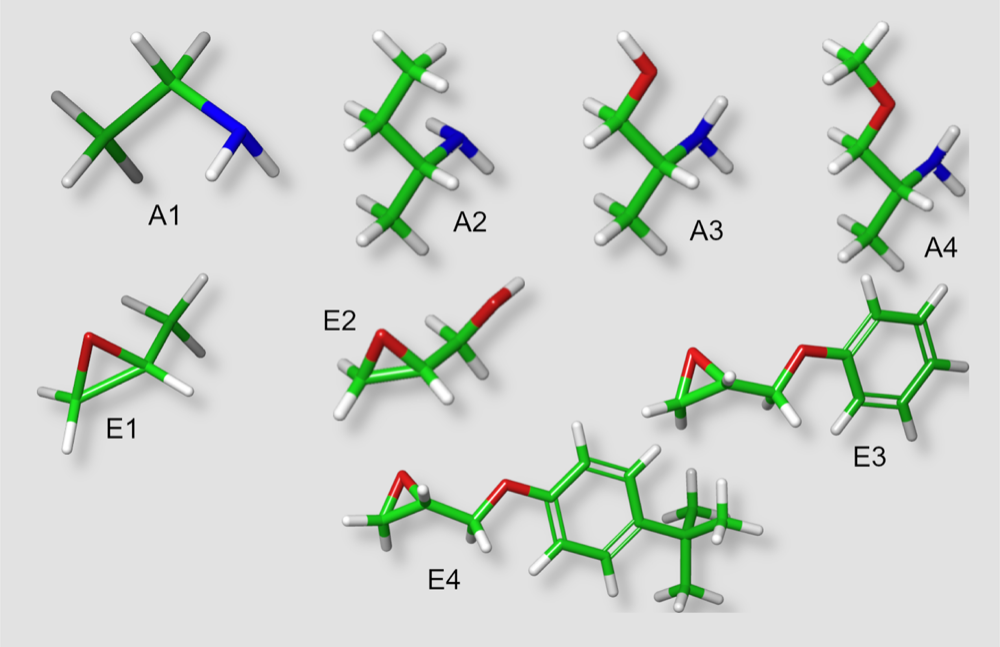
Cut-off structures for amine (A1–A4) and epoxy (E1–E4). The element coloring C: green, N: blue, O: red, and H: white, will be used throughout the study.

The role of the structural cutoffs was explored using reaction workflows recently introduced to the Schrödinger Materials Science Suite. These simulations involve freezing the primary reactive groups of the structures and swapping fragments from the rest of the structure based on pregenerated examples shown in Figure 1. Ethylamine was selected as A1, instead of the simpler methylamine, to reduce the otherwise expected significant contribution of the α-effect for the reference structure. Ethylamine was also used as the catalyzing amine for all relevant simulations. Similarly, isopropyl alcohol was used as the source of the catalyzing hydroxyl group. In the figure (Figure 1), carbons are colored green, nitrogens blue, oxygens red, and hydrogens white. The same coloring will be used throughout this study.

In order to find the most probable reactive paths, a conformational search—using the MacroModel program with the OPLS3e force-field^23^ for Monte Carlo Multiple Minimum searches—was added to each transition state search and reaction workflow. For the transition state searches only the lowest energy conformers are available as outputs, whereas in the reaction workflows ten lowest energy conformers are requested from separate conformational search runs the same starting structure. The final structural optimizations in DFT were performed using the LACVP** basis set, which—as the workflow default—resulted in significantly less convergence issues in the workflow than the 6-31G** basis set.

The final energies of each structure—including the outputs of the transition state searches—were calculated as SPE computations using the cc-pVTZ(-F) triple zeta basis set for improved accuracy. Other relevant parameters of the Jaguar code include grid density for the DFT computations (fine for SPE, medium for all other computations) and the accuracy of the pseudospectral computations (ultrafine grids with tight cutoffs for SPE, mixed grid with loose cutoffs for all other computations).

All results are reported as averages and standard deviations of grouped sets of these computational results. The conformers are grouped based on the used fragments (Figure 1) and subgrouped based on conformational similarities, for example, similar H-bonding states. The computation times are reported from SPE computations. Because the actual duration of the computation depends on the computational performance of the hardware and, for example, the use of parallelization to speed up the computation, the values are reported relative to the fastest computation (A1,E1 uncatalyzed primary amine reaction) and are based on the total CPU time.

## 3 Results and Discussion

**Figure 2:**
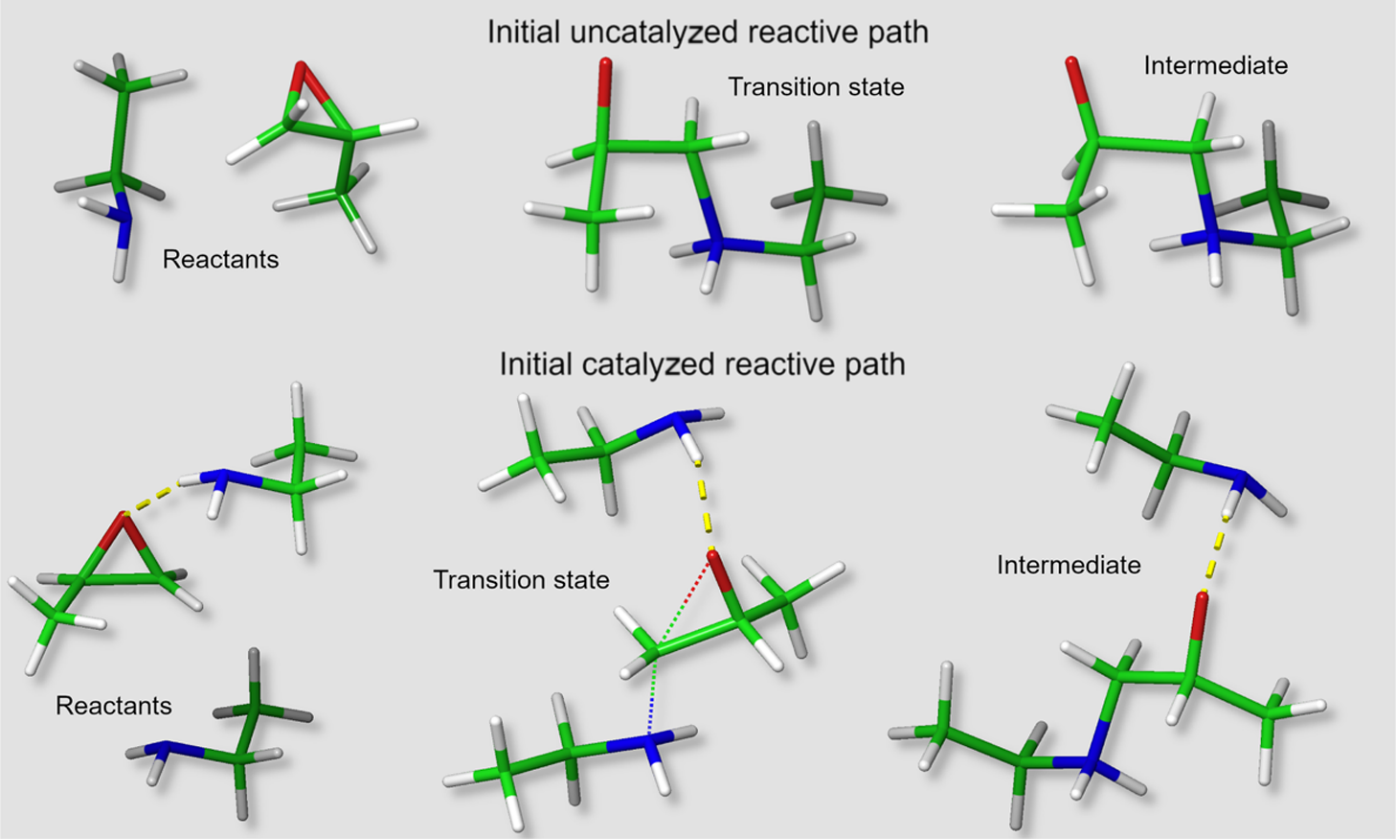
Reactive paths for simple and catalyzed primary addition reactions (A1,E1). The yellow dotted lines represent software identified H-bonds.

The computations begin with a transition state search for the simple A1,E1 system. This was needed both to find the reference energy levels for the simplified system and for use as an input for the reaction workflow computations. Figure 2 presents the results of the transition state searches used to find the primary amine addition reactive paths, including the A1,E1 version of the amine-catalyzed reactive path. These act as starting points for the rest of the computations.

The results for uncatalyzed reactions are presented in Table 1 as transition state energies relative to the lowest reactant structure energy. This relative energy is used as a measure for the activation energy (*E*_a_) or reactive barrier height.

**Table 1:** Activation Energies (*E*_a_) and the Number of TS Conformers (n) for the Uncatalyzed Reactive Paths^a^

structure	*E*_a_, [kcal/mol]		
– subgroup	primary (n)	secondary (n)	figure examples
A1, E1	34.26 (1)	32.31 (1)	Figure 2
– no interactions	34.26 (1)	32.31 (1)	
A2, E1	35.81 ± 0.72 (9)	39.75 ± 1.17 (10)	
– no interactions	35.81 ± 0.72 (9)	39.75 ± 1.17 (10)	
A3, E1	40.31 ± 1.55 (10)	41.03 ± 2.04 (9)	
– no interactions	41.47 ± 0.36 (6/10)	41.81 ± 1.70 (6/9)	
– Scheme 4	38.56 ± 0.39 (4/10)	40.58 ± 0.81 (2/9)	Figure 3a
– Scheme 2a	– (0)	37.21 (1/9)	Figure 3f,h
A4, E1	36.30 ± 1.67 (9)	36.81 ± 2.00 (10)	
– no interactions	36.30 ± 1.67 (9/9)	37.63 ± 1.09 (7/9)	
– Scheme 2a	– (0)	33.50 ± 0.66 (2/10)	Figure 3f,h
A1, E2	35.25 ± 1.39 (7)	35.46 ± 2.47 (9)	
– no interactions	35.25 ± 1.39 (7/7)	35.46 ± 2.47 (9)	
A1, E3	28.14 ± 0.77 (9)	30.48 ± 1.54 (9)	
– no interactions	28.88 ± 0.42 (3/9)	– (0)	
– Scheme 1b	27.77 ± 0.62 (6/9)	32.00 ± 0.48 (3/9)	Figure 3d,g,h
– Scheme 3b	– (0)	31.13 ± 0.21 (2/9)	Figure 4
– Scheme 3a	– (0)	29.01 ± 0.77 (4/9)	Figure 4
A1, E4	27.84 ± 0.51 (9)	29.66 ± 1.81 (9)	
– no interactions	28.27 ± 0.24 (3/9)	– (0)	
Scheme 1b	27.63 ± 0.47 (6/9)	30.27 ± 1.58 (4/9)	Figure 3d,g,h
– Scheme 3b	– (0)	31.30 ± 0.15 (2/9)	Figure 4
– Scheme 3a	– (0)	27.75 ± 0.66 (3/9)	Figure 4
A4, E4	27.14 ± 0.61 (10)	32.86 ± 1.68 (10)	
– Scheme 1	27.31 ± 0.57 (7/10)	33.51 ± 1.03 (6/10)	Figure 3d,g,h
–Schemes 1 and 2	– (0)	28.80 (1/10)	Figure 3h
– Schemes 1 and 4	26.72 ± 0.57 (3/10)	– (0)	Figure 3e
– Schemes 1 and 3b	– (0)	33.11 ± 0.81 (2/10)	Figure 4
– Schemes 1 and 3a	– (0)	32.51 (1/10)	Figure 4

a The conformers are subgrouped based on notable common interactions in Schemes 1–4. The “No Interactions” label indicates none of the presented schemes apply but other interactions with lesser impact on the reactivity can still be present.

The β-methylated hardeners (A2–A4) are known to result in slightly slower reactive systems compared to their linear counterparts. This is caused by the steric hindrance from the methyl group next to the amine.^14^ The effect is overlooked by the simplified reactive system (A1,E1), but clearly evident as higher activation energies for each larger amine structure (A2–A4,E1). This already highlights the crucial role of the selection of the cutoffs when studying specific reactive systems.

For the more complex structures, certain specific noncovalent interactions emerge and—despite the vast number of possible conformations for the rest of the molecule—the structures involving these interactions share structural and energetic similarities. To highlight these specific interactions, they are presented in Schemes 1–3.

**Scheme 1:**
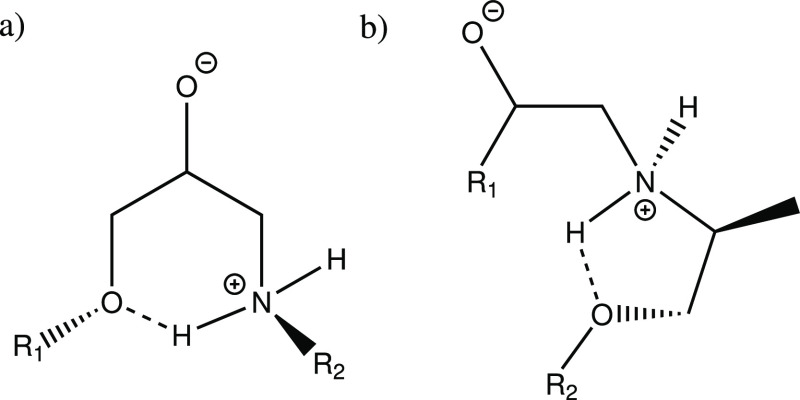
H-Bond between the Amine Group and a Backbone Oxygen from (a) Epoxy and (b) Amine. R_1_ Epoxy Backbone; R_2_ Amine Backbone

**Scheme 2:**
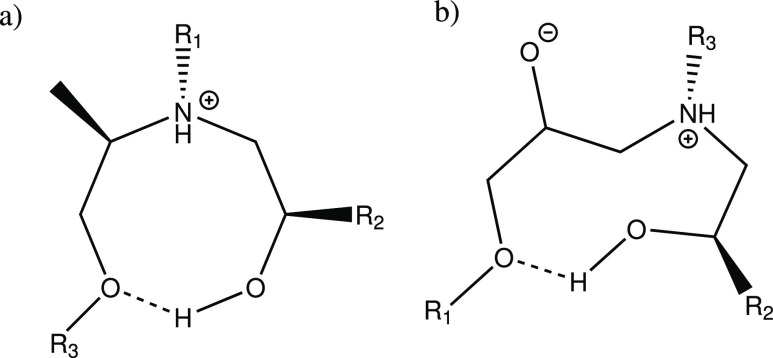
H-Bond between the Hydroxyl Group and a Backbone Oxygen from (a) Amine and (b) Epoxy R_1_ = epoxy backbone, R_2_ = epoxy backbone from primary amine reaction, R_3_ = amine backbone.

**Scheme 3:**
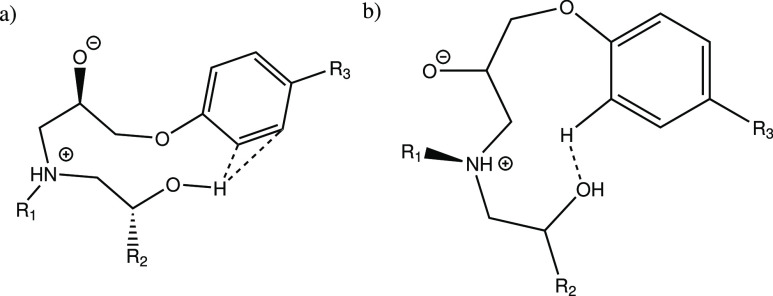
Noncovalent Interactions Involving the Aromatic Ring (a) Aromatic ring as a H-bond acceptor, (b) aromatic ring as a H-bond donor. R_1_ = amine backbone; R_2_ = epoxy backbone from primary amine reaction.

**Figure 3:**
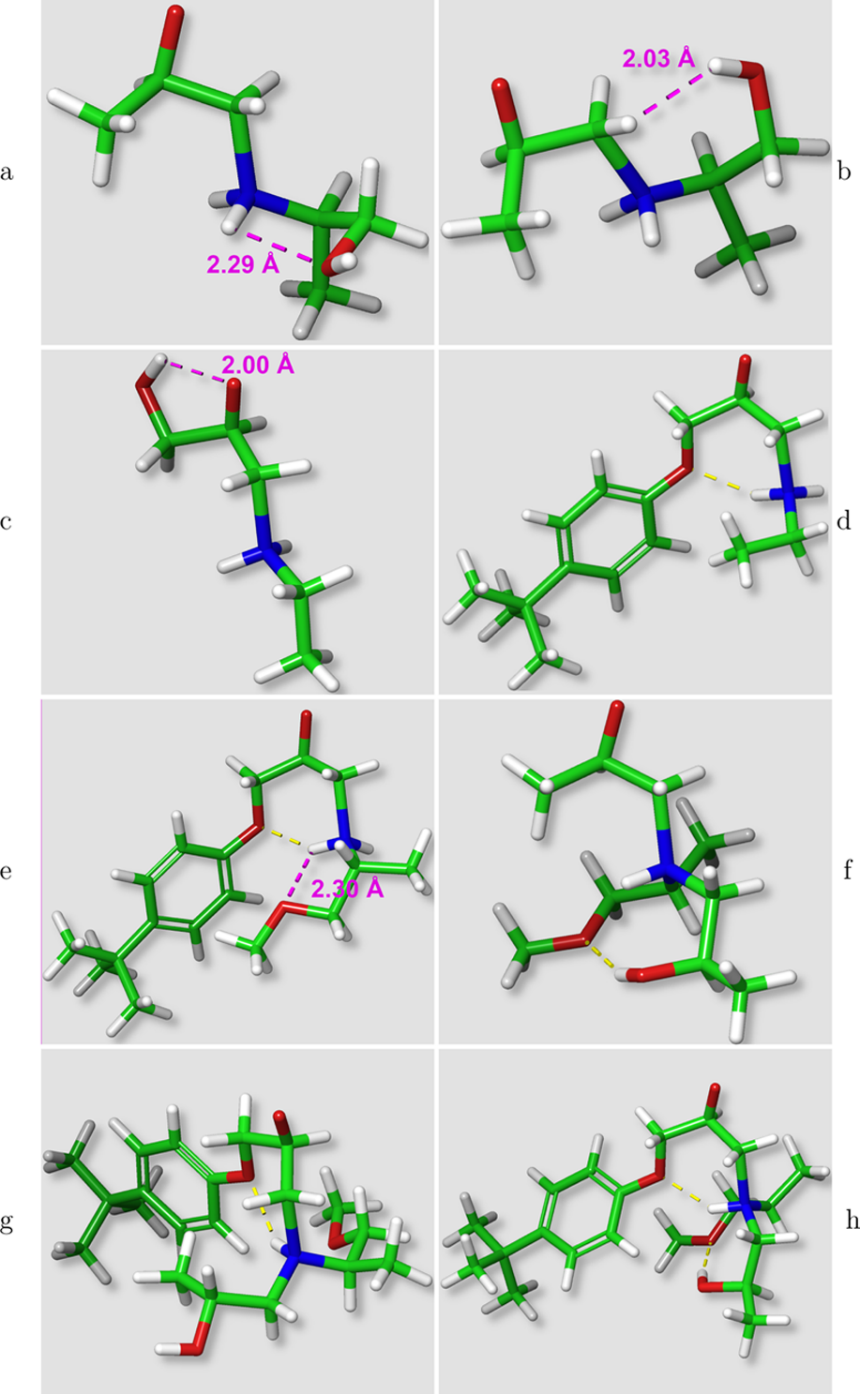
Uncatalyzed transition state conformers of (a,b) A3,E1, (c) A1,E2, (d) A1,E4, and (e) A4,E4 (primary) and (f) A4,E1, (g) A4,E4, and (h) A4,E4 (secondary). The yellow dotted lines represent software identified H-bonds.

**Scheme 4:**
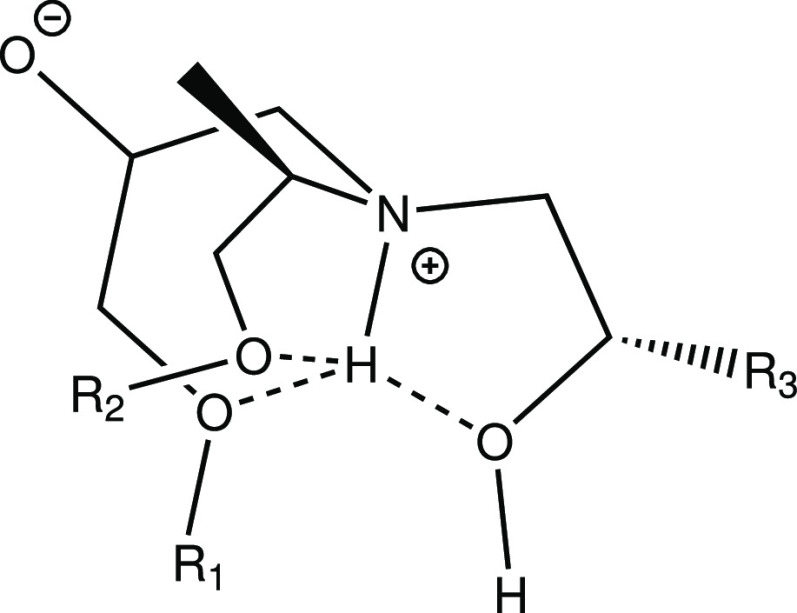
Complex Created from Multiple Surrounding O-groups Coordinating around the Amine Hydrogen. R_1_ = Epoxy Backbone, R_2_ = Amine Backbone, and R_3_ = Epoxy Backbone from Primary Amine Reaction

Contributions to the total energy can also be noted from conformations where the presence of H-bonding is uncertain but the proximity creates a more favorable energetic state. This is most common, for example, between an amine hydrogen and the nearby backbone oxygen (see, e.g., Figure 3a). Although similar to Scheme 1b, for larger structures, these often present as conformers with multiple oxygen groups coordinating around the amine hydrogen, as presented in 3e, Schemes 2a and 3. This type of complex is presented in Scheme 4.

The hydroxyl group in the structure E2 manifested mostly conformations where the hydroxyl group interacts with the epoxide oxygen (See Figure 3c). Although accurate for the structure in question, these conformations are considered bad for the purposes of exploring the reactivity of a DGEBA-based epoxy system, in which the oxygen is part of the epoxy backbone (see e.g. Figure 3d,e). The A3 hydroxyl group also manifested some conformations that would be very unfavorable in a larger system (Figure 3b) at least with the assumption that this oxygen is similarly part of the amine backbone. Due to these considerations the choice was made to exclude these systems from the computations of the catalyzed systems.

**Figure 4:**
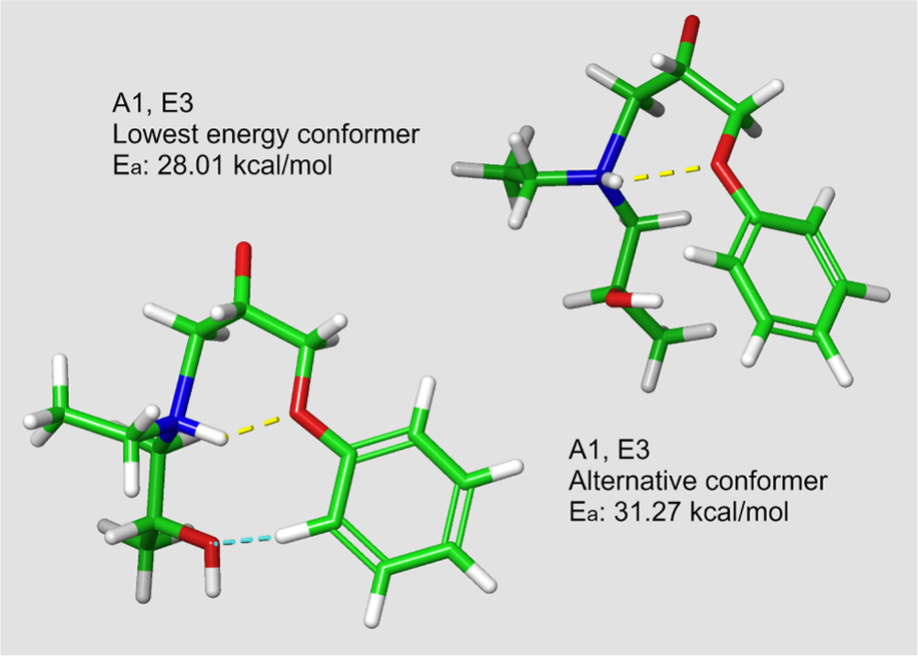
Different aromatic interactions in the A1,E3 secondary amine reaction. The yellow and blue dotted lines represent software identified H-bonds and aromatic interactions, respectively.

Interactions are also observed between the aromatic ring and the polar functional groups. First examples of this type of behavior are observable from the A1,E3 secondary amine reaction, where the average height of barrier changes approximately 1–2 kcal/mol depending on the type of interaction. The highest reactive barriers (32.00 ± 0.48 kcal/mol) are observed for the conformers with no interactions between the hydroxyl group from the primary amine reaction and the aromatic ring in the epoxy structure. For a PhH → OH type interaction (Scheme 3b), the average barrier height decreases to approximately 31.13 ± 0.21 kcal/mol. Aromatic H-bonds are more commonly reported between an H-bond donor and the aromatic ring.^24^ It is worth noting that the aromatic hydrogen in ortho-position to an oxygen is expected to have a slightly more acidic nature compared to a benzene hydrogen, which makes such interactions plausible. The lowest energy conformers (average of 29.01 ± 0.77 kcal/mol) present a OH → Ph type interaction (Scheme 3a) resembling the aromatic H-bond discussed by Brinkley and Gupta.^24^ The examples of such structures are presented in Figure 4. Similar interactions were observed in the uncatalyzed A4,E4 secondary amine system.

**Scheme 5:**
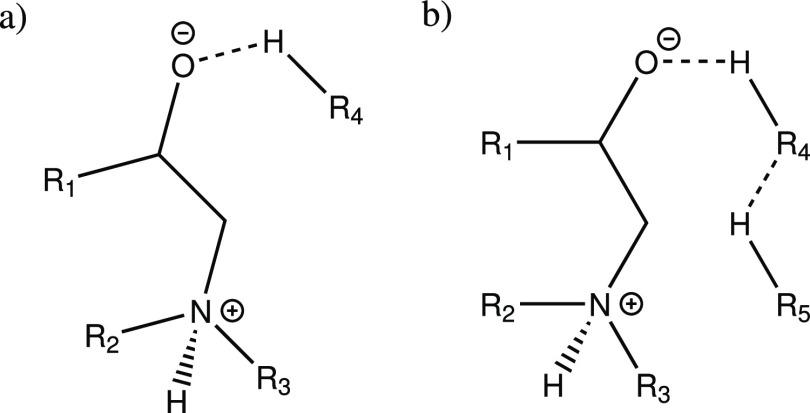
Catalysis of the Epoxy-Amine Reaction by an Extra functional Group (OH or NH_2_ = R_4_) R_1_ = epoxy backbone, R_2_ = amine backbone, R_3_ = either hydrogen or the epoxy from primary amine reaction. In the models presented here R_5_ is connected to one of R_1-3_.

When functional groups (amine or hydroxyl) are added to catalyze the reaction, the H-bonding of these added functional groups clearly changes the energetics of the reaction, which is in line with existing studies on the topic.^11,15^ The catalysis is based on the extra functional group interacting with the opening epoxide ring, as presented in Scheme 5.

Mobility around the reactive center is likely overestimated in such a small model system, that is, it is improbable that every reactive site would have sufficient free volume surrounding it to allow this type of catalysis. Nevertheless, the model still helps highlight the differences between different options for H-bond formation, namely the hydroxyl and amine groups, and helps estimate the role of such catalysis in total reactivity. The transition state energies for the catalyzed reaction paths are presented in Table 2.

**Table 2:** Overview of the Relative Barrier Heights for the Different Structural Cutoffs for the Catalyzed Cases^a^

structure	*E*_a_, amine-cat. [kcal/mol]		*E*_a_, hydroxyl-cat. [kcal/mol]	
– subgroup	primary	secondary	primary	secondary
A1, E1	20.61	34.55 ± 2.90	32.22 ± 3.28	32.18 ± 1.48
– no catalysis			41.52	
– Scheme 5a	20.61	36.30 ± 0.61	31.18 ± 0.16	32.56 ± 0.87
– Scheme 5b		30.48 ± 0.97		28.69
A2, E1	31.00 ± 0.78	32.20 ± 3.24	31.24 ± 0.66	31.67 ± 2.75
– no catalysis	31.33 ± 0.29			
– Scheme 5a	30.67 ± 0.99	35.19 ± 0.64	31.24 ± 0.66	33.28 ± 1.54
– Scheme 5b		29.21 ± 0.90		28.43 ± 0.91
A4, E1	30.14 ± 1.45	31.33 ± 3.43	29.38 ± 1.65	32.94 ± 1.67
– no catalysis		28.47		
– Scheme 5a	30.14 ± 1.45	34.68 ± 1.61	29.38 ± 1.65	32.98 ± 0.79
– Schemes 2a and 5a		32.23		32.96
– Scheme 5b		28.48 ± 2.39		29.85 ± 0.28
A1, E3	25.06 ± 0.69	26.40 ± 3.44	22.15 ± 0.50	26.09 ± 2.35
– no catalysis	24.66 ± 0.60			
– Scheme 5a		28.74 ± 0.71		
– Schemes 1a and 5a	25.66 ± 0.13	28.27 ± 1.60	22.09 ± 0.48	27.15 ± 1.31
– Schemes 1a and 5b		21.71 ± 0.69		22.35 ± 0.63
– Scheme 5b			22.68	24.67
– Schemes 3a and 5a				27.90 ± 0.21
A1, E4	26.76 ± 0.78	28.45 ± 3.58	23.69 ± 0.82	25.67 ± 1.46
– Scheme 5a	26.10 ± 0.52	31.46 ± 0.94		
– Schemes 1a and 5a	27.09 ± 0.67	28.94 ± 2.14	24.00 ± 0.88	25.69 ± 1.23
– Schemes 1a and 5b		22.68 ± 0.26		27.67
– Scheme 5b			23.22 ± 0.50	23.56
A4, E4	25.24 ± 1.50	32.06 ± 4.67	24.16 ± 1.22	24.45 ± 1.64
– Schemes 1 and 5a	25.24 ± 1.50	37.16 ± 3.97	24.32 ± 1.20	25.56 ± 0.01
– Scheme 5a		31.37 ± 1.46		
– Schemes 1, 3b and 5a		31.69		25.45 ± 0.63
– Schemes 3a, 4 and 5a				21.94 ± 0.08
– Scheme 5b			22.88	
– Schemes 1, 3b and 5b		26.81 ± 4.46		
– Schemes 1 and 5b		29.65		
– Schemes 3a and 5b				24.30

a The conformers are subgrouped based on notable common interactions in Schemes 1–5. “No catalysis” indicates conformations with no predicted interaction between the catalyzing functional group and the opening epoxide ring.

**Figure 5:**
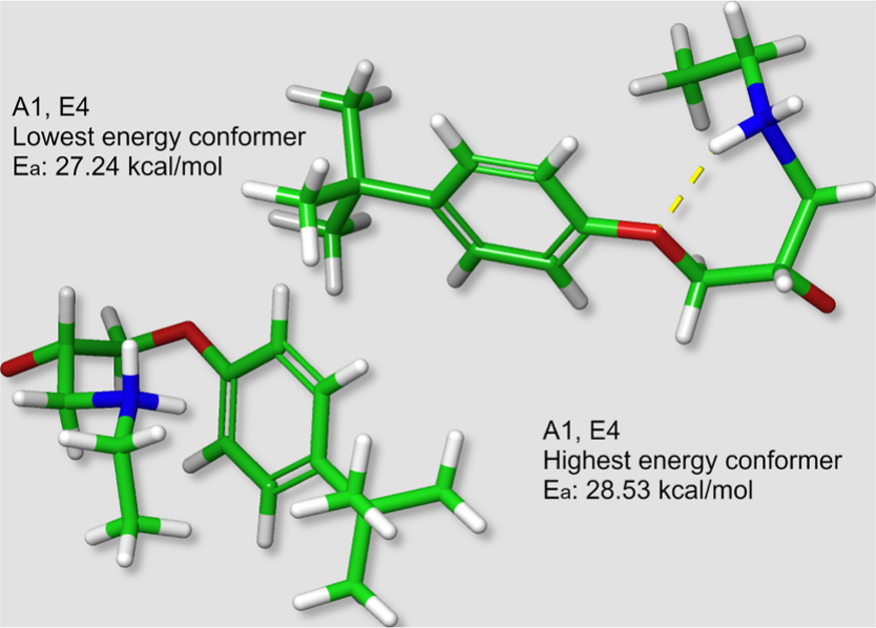
Maximum and minimum energy conformers for the A1,E4 primary amine reaction. The yellow dotted lines represent software identified H-bonds.

The H-bond between the amine group and a backbone oxygen (Scheme 1) appears a favorable conformation for systems large enough to orient suitably. Figure 5 shows examples of the effect this interaction has on the energy in the A1,E4 system. The effect on barrier height is unexpectedly minor. A possible explanation is that the orientation forces some bond angles to deviate from their equilibrium values increasing the energy.

**Figure 6:**
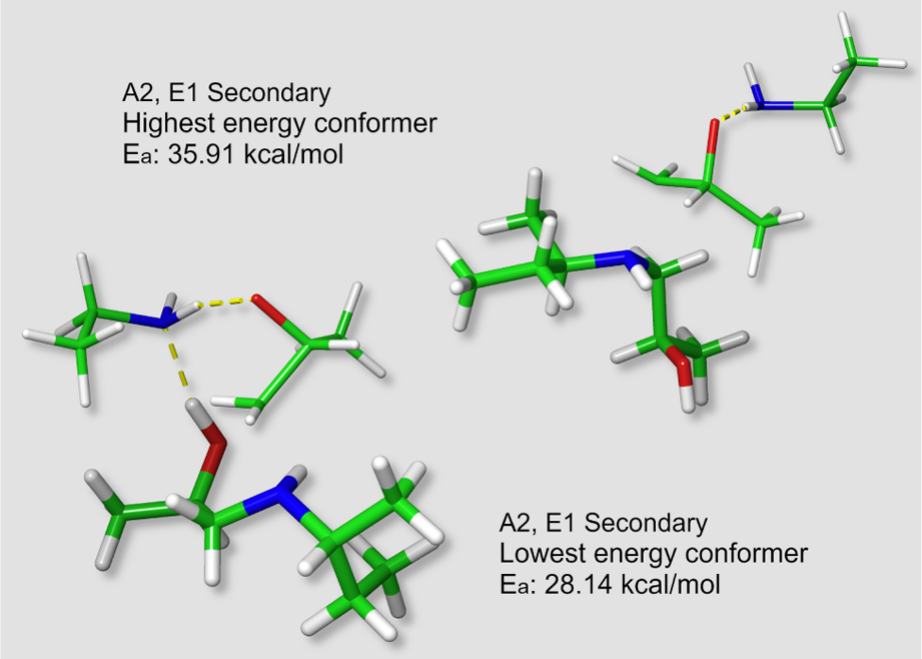
Maximum and minimum energy conformers for the amine-catalyzed A2,E1 secondary amine reaction. The yellow dotted lines represent software identified H-bonds.

Figure 6 shows the lowest and highest energy conformers for the amine-catalyzed A2,E1 secondary amine transition states. A complex H-bond network formed between the hydroxyl group—created in the primary amine reaction—and the amine-catalyzed reactive core (Scheme 5b) significantly lowers the reactive barrier for this path. It is also worth noting that the required orientation of the hydroxyl group also brings it close to the remaining amine hydrogen. The combined effect is quite significant (29.21 ± 0.90 kcal/mol VS 35.19 ± 0.64 kcal/mol).

**Figure 7:**
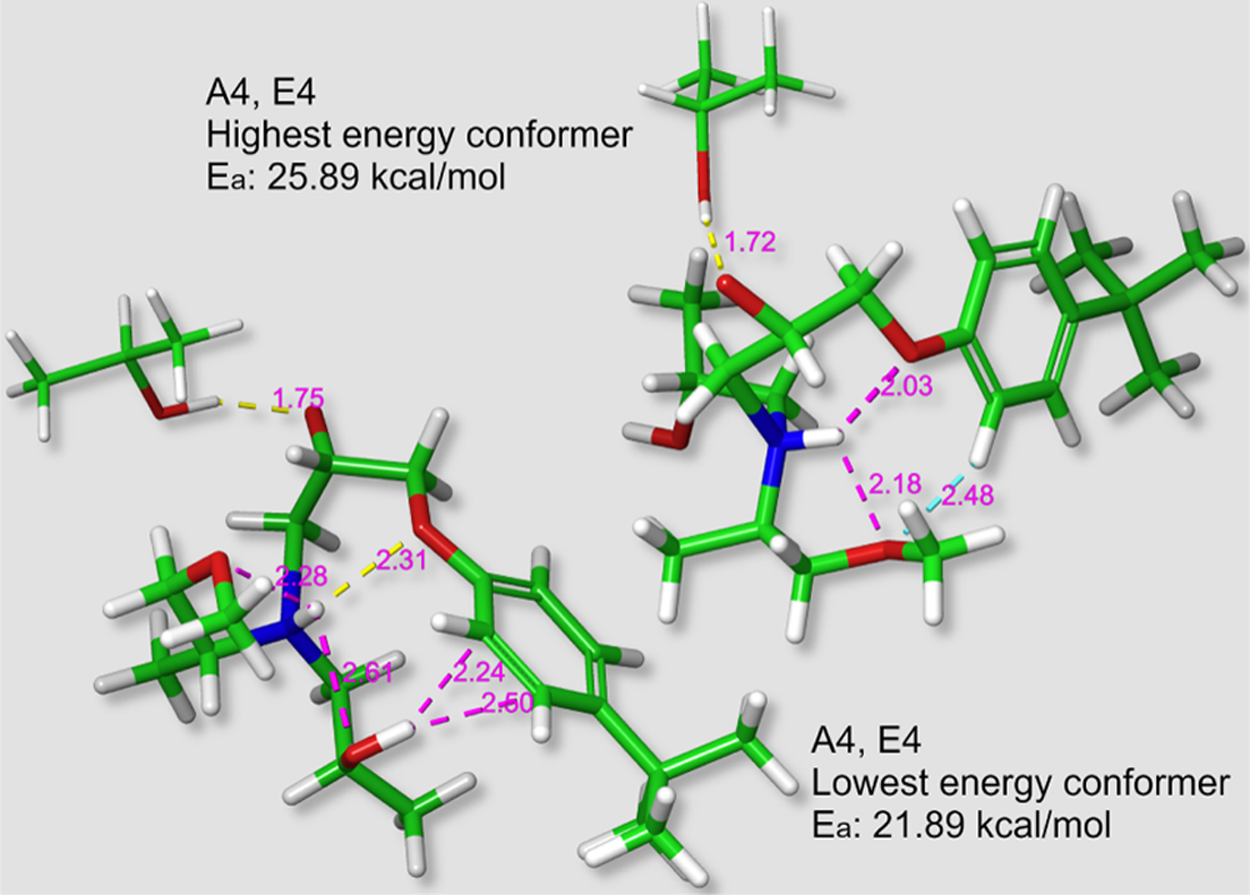
Maximum and minimum energy conformers for the hydroxyl-catalyzed A4,E4 secondary amine reaction. The yellow, blue, and magenta dotted lines represent software identified H-bonds, aromatic interactions, and the measured interatomic distances, respectively.

In general, for the larger systems, the conformations and their interpretation become challenging due to the number of available interactions including possible complex cases such as the one presented in Scheme 4. For example, in the catalyzed A4,E4 systems, multiple noncovalent interactions are noted for most conformers (see Figure 7). The lowest energy conformations show oxygens coordinating around the amine hydrogen as in Scheme 4. In the higher energy conformers, however, either the amine backbone oxygen or the hydroxyl group from the primary amine is oriented away from the amine hydrogen. The difference in energies between these conformations appears to be in the range of 2–4 kcal/mol but in all cases other interactions contribute. A similar coordination around the amine hydrogen can also be observed in the uncatalyzed system presented in Figure 3h, and a similar difference in energy is observed when one of the oxygens is oriented less favorably (see Figure 3g). Also, again additional interactions contribute, such as the OH → O H-bond presented in Figure 3h.

The aromatic interactions contribute significantly also in the catalyzed models. With the reactive center largely the same for a majority of the structures, the trend of increasing deviations for larger systems can be often attributed to how, for example, the hydroxyl group formed in the primary amine reaction is oriented in relation to the aromatic ring in the secondary amine reactions. The strength of the interaction—for cases resembling Scheme 3a—is in these results similar to what is observed for H-bonding, giving validity to the term of aromatic H-bonding.

Predicting the actual importance of the catalyzed paths using the DFT computation is difficult as the systems are effectively considered infinitely diluted, whereas molecular motion in the real epoxy-amine reactive systems is far more restricted. The observed catalyzing effect partly explains the accelerated reactivity when amine hardener is added in excess of the stoichiometric ratio. It is also very likely that whatever importance the catalysis has, it is mitigated as the curing process progresses, and the required molecular motions are restricted until it—after vitrification—become practically nonexistent.

Table 3 presents an overview of the computational times—the easiest measure for the computational cost—for the different systems. Some interesting observations can be made from these results. The most obvious one is as follows: increasing the complexity of the system increases the computational cost but also variation of the computational time. In the SPE computation, the biggest source of variation between similar structures appears to be the dispersion correction steps in the computation. The slightly longer computational times for hydroxyl-catalyzed compared to amine-catalyzed systems are likely a result of the slightly bigger system (isopropanol vs ethylamine).

**Table 3:** Relative Computation Times for Different System Sizes (Single SPE Computation)^a^

	uncatalyzed		amine		hydroxyl	
structure	primary	secondary	primary	secondary	primary	secondary
A1, E1	1.00 ± 0.09	3.61 ± 0.44	4.05 ± 0.43	6.43 ± 2.07	4.89 ± 0.31	11.4 ± 1.4
A2, E1	2.93 ± 0.29	6.09 ± 0.92	7.60 ± 1.54	11.5 ± 2.6	9.76 ± 1.56	19.3 ± 3.1
A3, E1	2.18 ± 0.12	5.60 ± 0.54				
A4, E1	2.61 ± 0.29	4.97 ± 0.47	8.85 ± 1.17	17.4 ± 3.1	10.3 ± 2.4	20.4 ± 3.7
A1, E2	1.55 ± 0.22	4.47 ± 0.66				
A1, E3	6.71 ± 0.83	10.8 ± 1.7	12.0 ± 3.9	26.1 ± 5.7	13.9 ± 4.0	24.0 ± 7.5
A1, E4	14.6 ± 1.9	17.9 ± 3.0	20.0 ± 5.3	35.0 ± 11.6	22.5 ± 7.0	40.6 ± 9.4
A4, E4	18.6 ± 1.7	35.5 ± 4.4	24.6 ± 3.4	35.3 ± 3.7	42.8 ± 10.1	38.4 ± 3.6

a The reference time is approximately 2332 cpu seconds.

The reactant conformations of the reaction workflows provide an interesting point of discussion. Especially for the catalyzed reactive paths the lowest energy conformers show significant interactions between the added catalyzing functional group (OH or NH) and either one or both of the amine and epoxy functional sites. These minimal energy conformers tend to orient unfavorably considering the reactive path of the epoxy-amine addition reaction. Whether the needed reorientation manifests as an initial smaller reactive barrier, could be an interesting point of further study. However, the effect is likely small compared to the advantage offered by the lowering of the reactive barrier in the rate determining step studied here.

No conclusive retardation effect was observed based on the computation results. However, some hints at possible metastable intermediate structures can be drawn from the results of the conformational searches of the intermediate structures. For example, the uncatalyzed primary amine reaction workflows resulted in intermediate structures close to the final product—indicating no local minima near the transition state. Whereas for the secondary amine reaction, the conformational search converged to local minima structurally and energetically near the transition state for all systems larger than the A1,E1 system. This could indicate the secondary amine reaction for our model system is in fact slower, even though the activation energies are largely similar. Here, as the core reactive path was kept largely constant in the set-up of the computations, possible key considerations, such as a backbone oxygen interacting and “deactivating” the catalyzing functional group, were not explored.

## 4 Conclusions

In this study, the epoxy-amine reaction was studied using the DFT computations at different cut-off scales to study the role various noncovalent interactions of the neighboring functional groups. A secondary goal was to test the potential of such computations in predicting the reactivity of a specific epoxy-amine system, at least qualitatively, with promising results. The selected cutoffs significantly affect the final energy levels. Most consistent results were achieved with the largest and the smallest cut-off levels. The energy levels for the smallest and the largest structures are also very similar, which should be coincidental. Various important interactions were included in the computations as a result of the increased complexity of the systems. Based on our computations, the order of importance for different types of interactions to epoxy-amine reactivity is as follows: intermolecular H-bonding, intramolecular H-bonding, aromatic interactions, and inductive effects, from most important to least important. Computations with the smallest cutoffs (largest structures) unveil the possible effects of the chemical groups near the reactive site that can stabilize the transition state or lower the energy level and accelerate the reaction. However, these overlapping phenomena proved to be quite challenging to analyze. Expanding the size of a reactive system in DFT computations must be approached with care. A simulation more representative of your real system can offer insights into the behavior of the system. The drawbacks are losing generality and, of course, a significant increase in computational cost..

## Supporting Information Available

All final structures in Maestro.mae format (ZIP)
Overview of the comparative trials with other functionals than B3LYP-D3 (PDF)
