# Supplementary material for: Cut-off Scale and Complex Formation in Density Functional Theory Computations of Epoxy-Amine Reactivity
Source: ACS Omega. 2021 Oct 25;6(44):29424–31. doi: 10.1021/acsomega.1c03229 (PMC8587129; doi:10.1021/acsomega.1c03229)
Supplement: Supplementary file 2 — ao1c03229_si_002.pdf [file ao1c03229_si_002.pdf]

Supporting Information

Cut-off scale and complex formation in DFT computations of epoxy-amine reactivity

Pekka V. Laurikainen*[,1], Essi L. Sarlin[1]

[1]Tampere University, Faculty of Engineering and Natural Sciences, P.O. Box 589, FI-33014 Tampere University, Finland.

1. Final geometries

The Maestro files containing the final computational results of this work are available free of charge on the ACS Publications website https://pubs.acs.org/ (ZIP).

To open the files, you need a software capable of opening Maestro .mae files. For academic users the simplest solution is downloading the free Maestro visualization program from Schrödinger Inc. available at https://www.schrodinger.com/freemaestro .

In addition to the Maestro software, at least VMD (https://www.ks.uiuc.edu/Research/vmd/) recognizes the Maestro file format.

2. Other functionals

As described in the main text, selected computations were also tested with other functionals that could be suitable for the task. This was done solely for the purpose to support the selection of B3LYP-D3.

The M06-2X and ωB97x-D functionals ware tested with SPE computation of the outputs of the conformational search workflow. It is worth noting again that the workflow included a DFT structural optimization step done with LACVP** basis and the B3LYP-D3 functional. The results of the M06-2X computations gave similar results for the relative energy levels of the structures but the energy levels themselves were vastly different. The gas phase energies from the computation with the M06-2X functional were on average 281.58 ± 3.28 kcal/mol higher. The average computation time for the A4, E1 system was approximately 89700 CPU seconds vs. approximately 47500 CPU seconds with B3LYP-D3. The corresponding computations with the ωB97x-D functional failed to converge.

Becke-Johnson damping has been shown to improve the predictions of non-covalent interactions of D3 dispersion corrected DFT [1,2]. This modification was available in the used software as a functional denoted B3LYP-D3(BJ). Our test computations for the A4, E1 system give very similar results for the two variations. With the Becke-Johnson damping, the gas phase energies are on average 16.02 ± 0.67 kcal/mol lower but the results are otherwise practically identical. In terms of performance, it seems B3LYP-D3(BJ) slightly out-performs B3LYP-D3 with an average computation time of approximately 40500 CPU seconds. The results are however similar enough to not invalidate the original selection B3LYP-D3.

* Corresponding Author
email: pekka.laurikainen@tuni.fi

References

1. Grimme, S.; Ehrlich, S.; Goerigk, L. Effect of the damping function in dispersion corrected density functional theory. *Journal of Computational Chemistry* **2011** *32* (7), 1456–1465, DOI: 10.1002/jcc.21759.
2. Daniel G. A. Smith, Lori A. Burns, Konrad Patkowski, and C. David Sherrill. Revised Damping Parameters for the D3 Dispersion Correction to Density Functional Theory. *The Journal of Physical Chemistry Letters* **2016** *7* (12), 2197-2203 , DOI: 10.1021/acs.jpclett.6b00780.
